# Recovery of left ventricular dysfunction after ST-elevation myocardial infarction: comparison between 2D Doppler echocardiography and contrast enhanced cardiac MRI

**DOI:** 10.1186/1532-429X-13-S1-P120

**Published:** 2011-02-02

**Authors:** Daniel M Couri, Matthew W Martinez, Imran S Syed, Scott Harris, James Glockner, Phillip A Aroaz, Abhiram Prasad, Charanjit S Rihal, Jae K Oh

**Affiliations:** 1 Mayo Clinic, Springfield, IL, USA; 2 Le High Valley Health Network, Allentown, PA, USA; 3 Mayo Clinic, Rochester, MN, USA; 4 Memorial University, Newfoundland and Labrador, NL, Canada

## Background

The role of contrast enhanced cardiac MRI (ceMRI) in predicting recovery of left ventricular dysfunction is evolving; yet transthoracic echocardiography (TTE) has not been evaluated in this manner.

## Methods

Patients with acute ST-elevation MI (STEMI) admitted to Mayo Clinic from August, 2007 to September, 2008 who received reperfusion therapy were screened with TTE and ceMRI. Patients with ceMRI evidence of a previous MI were excluded. Left ventricular dysfunction was defined as an ejection fraction (LVEF) < 50%.

## Results

Of 116 patients screened, 91 met inclusion criteria and 56 patients completed follow-up imaging (3-79 weeks). 89% of both the imaging studies were completed within 3 days of the index MI and 32 (57%) patients had a baseline LVEF <50%. The ability of ceMRI and TTE variables to predict normalization of LVEF was assessed using ROC analysis. Delayed enhancement left ventricular mass index (DEi) (DEi <20% 35/56 (63%) was the strongest (AUC 0.86, p=0.0004) predictor of LVEF recovery with a sensitivity, specificity, positive predictive value, and negative predictive value of 0.79, 0.92, 0.94, and 0.75 respectively. When combined with DEi, wall motion score index (WMSI) significantly correlated with follow-up EF (r=0.81, p<0.0001) and overall change in EF (r=0.73, p<0.001). Acute measures of diastolic function did not meaningfully predict LVEF recovery.

## Conclusions

A DEi of <20%, as assessed by ceMRI, is the strongest predictor of LVEF normalization in patients with acute post-STEMI left ventricular dysfunction. As compared with TTE, ceMRI provides uniquely valuable information for the long-term management of post-STEMI patients.

